# R497K polymorphism in epidermal growth factor receptor gene is associated with the risk of acute coronary syndrome

**DOI:** 10.1186/1471-2350-9-74

**Published:** 2008-07-30

**Authors:** Lin-Bo Gao, Bin Zhou, Lin Zhang, Ye-Sheng Wei, Yan-Yun Wang, Wei-Bo Liang, Mei-Li Lv, Xin-Min Pan, Yu-Cheng Chen, Li Rao

**Affiliations:** 1 Department of Forensic Biology, West China School of Preclinical and Forensic Medicine, Sichuan University, Chengdu 610041, Sichuan, PR China; 2 Laboratory of Molecular Translational Medicine, West China Second University Hospital, Sichuan University, Chengdu 610041, Sichuan, PR China; 3 Department of Immunology, West China School of Preclinical and Forensic Medicine, Sichuan University, Chengdu 610041, Sichuan, PR China; 4 Department of Forensic Pathology, West China School of Preclinical and Forensic Medicine, Sichuan University, Chengdu 610041, Sichuan, PR China; 5 Department of Cardiology, West China Hospital of Sichuan University, Chengdu 610041, Sichuan, PR China

## Abstract

**Background:**

Previous studies suggested that genetic polymorphisms in the epidermal growth factor receptor (EGFR) gene had been implicated in the susceptibility to some tumors and inflammatory diseases. EGFR has been recently implicated in vascular pathophysiological processes associated with excessive remodeling and atherosclerosis. Acute coronary syndrome (ACS) is a clinical manifestation of preceding atherosclerosis. Our purpose was to investigate the association of the EGFR polymorphism with the risk of ACS. In this context, we analyzed the HER-1 R497K and EGFR intron 1 (CA)_n _repeat polymorphisms in 191 patients with ACS and 210 age- and sex-matched controls in a Chinese population, using a polymerase chain reaction-restriction fragment length polymorphism (PCR-RFLP) strategy and direct sequencing.

**Results:**

There were significant differences in the genotype and allele distribution of R497K polymorphism of the EGFR gene between cases and controls. The *Lys *allele had a significantly increased risk of ACS compared with the *Arg *allele (adjusted OR = 1.49, 95% CI: 1.12–1.98, adjusted *P *= 0.006). However, no significant relationship between the number of (CA)_n _repeats of EGFR intron 1 (both alleles < 20 or any allele ≥ 20) and the risk of ACS was observed (adjusted OR = 0.97, 95% CI: 0.58–1.64, adjusted *P *= 0.911). Considering these two polymorphisms together, there was no statistically significant difference between the two groups.

**Conclusion:**

R497K polymorphism of the EGFR gene is significantly associated with the risk of ACS. Our data suggests that R497K polymorphism may be used as a genetic susceptibility marker of the ACS.

## Background

Acute coronary syndrome (ACS) is a clinical manifestation of preceding atherosclerosis, inflammation and thrombosis [1]. It constitutes an important cause of mortality and morbidity in many countries including China. Although the exact mechanism of ACS pathogenesis is not fully known, many factors such as age, gender, smoking, hypertension, diabetes mellitus, hypercholesterolemia, obesity and sedentary lifestyle are involved in the disease [2-4]. In addition, several studies have shown that genetic factors such as single nucleotide polymorphisms (SNPs) of angiotensin converting enzyme (ACE), platelet glycoprotein IIb HPA-3, and matrix metalloproteinase-3 (MMP-3) also play a crucial role in the development of ACS [5-7]. Despite many gene SNPs [5-14] have been reported to be associated with ACS, it is necessary to discover novel gene variants in the risk of ACS. Firstly, ACS is a disease related to multiple genes. Secondly, the molecular mechanism of ACS is unclear, and it may be benefit to elucidate the pathogenesis of ACS by finding novel gene variants.

The epidermal growth factor receptor (EGFR) is a transmembrane glycoprotein of 170 kDa (1186 amino acids) encoded by a gene located in the short arm of chromosome 7 (chromosome 7p12.1–12.3) [15]. And it contains 26 exons [16]. A polymorphic variant of the EGFR arising from a single nucleotide change (G → A) leading to an arginine (*Arg*) →Lysine (*Lys*) substitution in codon 497 (HER-1 R497K) in the extracellular domain within subdomain IV of the EGFR gene has been identified [17]. It is one of the key polymorphisms within the EGFR signaling pathway. An in vitro study has shown that the variant HER-1 497K has attenuated functions in ligand binding, growth stimulation, tyrosine kinase activation, and induction of protooncogenes myc, fos, and jun compared with the "wild-type" HER-1 497R [18]. Another polymorphic variant of the EGFR is a highly polymorphic region in intron 1 of the EGFR gene which is associated with transcription levels of EGFR in vitro and in vivo [19,20]. The length of this (CA)_n _dinucleotide polymorphism correlates inversely with the transcriptional activity of the gene. *In vitro *studies have shown that the transcriptional activity in cell lines containing a prolonged polymorphic region (>20 CA repeats) was markedly reduced compared with cells containing a shorter allele (16 repeats). Recently, several studies reported that genetic polymorphisms of the EGFR gene had been implicated in the risk of a range of tumors [21-26]. In addition to its prominent role in the development of several cancers [27], EGFR has been currently implicated in vascular pathophysiological processes associated with excessive remodeling and atherosclerosis [28,29]. Moreover, EGFR has been identified on intimal smooth muscle cells within human atherosclerotic plaque and cultured rat aortic smooth muscle cells, which mediates cell proliferation and DNA synthesis [30-32]. Atherosclerosis, regarded as a chronic inflammatory process [33], plays a fundamental role in the pathogenesis of ACS.

Therefore, we hypothesized that HER-1 R497K and EGFR intron 1 (CA)_n _repeat polymorphisms may modulate the susceptibility to ACS. To test this hypothesis, we conducted a hospital-based case-control study to evaluate the potential association between HER-1 R497K and intron 1 (CA)_n _repeat polymorphisms in the EGFR gene and the risk of ACS in a Chinese population.

## Results

### Characteristics of the study population

The demographics of the cases and controls enrolled in this study are shown in Table 1. There were no significant differences between the cases and controls for the age, gender distribution and body mass index. However, the smoking status, total cholesterol, LDL-cholesterol and triglycerides were significantly higher in the ACS group than in the control group(*P *< 0.001), whereas HDL-cholesterol was significantly lower in the ACS group than in the control group(*P *< 0.001). Hypertension, diabetes mellitus and coronary stenosis were observed only in patients with ACS.

**Table 1:** The characteristics of the study population

	Control	ACS	*P*^a^
		
	n = 210 (%)	n = 191 (%)	
Sex (M/F)	132/78 (62.9/37.1)	112/79 (58.6/41.4)	NS
Age (years; mean ± SD)	59.4 ± 11.2	60.6 ± 10.9	NS
Body mass index (kg/m^2^; mean ± SD)	25.2 ± 3.3	26.6 ± 4.2	NS
UA/NSTEMI/STEMI		102/49/40(53.4/25.7/20.9)	
Smoking status (never/former/current)	111/53/46 (52.9/25.2/21.9)	62/73/56 (32.5/38.2/29.3)	< 0.001
Total cholesterol (mmol/l; mean ± SD)	3.57 ± 0.93	5.24 ± 1.19	< 0.001
HDL-cholesterol (mmol/l;mean ± SD)	1.15 ± 0.40	1.02 ± 0.42	< 0.001
LDL-cholesterol (mmol/l; mean ± SD)	2.21 ± 0.69	3.04 ± 0.99	< 0.001
Triglycerides (mmol/l; mean ± SD)	1.27 ± 0.55	1.92 ± 0.90	< 0.001
Hypertension		85 (44.5)	
Diabetes mellitus		38 (19.9)	
Number of stenosis vessels			
1		88 (46.1)	
2		75 (39.3)	
3		28 (14.7)	

ACS, acute coronary syndrome; UA, unstable angina; NSTEMI, non-ST-segment elevation myocardial infarction; STEMI, ST-segment elevation myocardial infarction; NS, not significant

^a ^ACS vs. controls by Student's t-test or Chi-squared test.

### HER-1 R497K polymorphism

The genotype and allele frequencies of the HER-1 R497K polymorphism between the controls and the cases are shown in Table 2. And the genotype distributions were in Hardy-Weinberg's equilibrium. There were significant differences in the genotype and allele frequency of the HER-1 R497K polymorphism between ACS and control groups. Compared with the *Arg *allele, the frequency of the *Lys *allele was significantly higher in the patient group than in the control group (adjusted OR = 1.49, 95% CI: 1.12–1.98, adjusted *P *= 0.006).

**Table 2:** The genotype and allele distribution of EGFR polymorphisms in ACS patients and controls

Polymorphism	Control n = 210 (%)	ACS n = 191 (%)	Crude OR (95% CI)	Adjusted OR^a ^ (95% CI)	Adjusted *P*^a^
R497K					
genotypes					
*Arg/Arg*	45 (21.4)	32 (16.8)	1.00 (Ref)	1.00 (Ref)	1.00 (Ref)
*Arg/Lys*	107 (51.0)	79 (41.4)	1.04 (0.61–1.78)	1.07 (0.62–1.85)	0.807
*Lys/Lys*	58 (27.6)	80 (41.9)	1.94 (1.10–3.41)	1.98 (1.12–3.49)	0.018
alleles					
*Arg *allele frequency	197 (46.9)	143 (37.4)	1.00 (Ref)	1.00 (Ref)	1.00 (Ref)
*Lys *allele frequency	223 (53.1)	239 (62.6)	1.48 (1.11–1.96)	1.49 (1.12–1.98)	0.006
(CA)_n _repeats					
Both (CA)_n _repeats < 20	35 (16.7)	33 (17.3)	1.00 (Ref)	1.00 (Ref)	1.00 (Ref)
Any (CA)_n _repeats ≥ 20	175 (83.3)	158 (82.7)	0.96 (0.57–1.61)	0.97 (0.58–1.64)	0.911

^a ^adjusted for sex and age by the logistic regression model.

### EGFR intron 1 (CA)n repeat polymorphism

In 420 chromosomes from 210 controls, 10 alleles with 14–23 CA repeats were found (Table 3). The allele distribution in healthy Chinese individuals, ranging from 0.5% to 42.9% with a predominance of 16 and 20 repeats, was in agreement with that of Asian populations reported in a previous study [34]. The allele distribution in ACS patients was similar to the control group except that a novel (CA)_n _repeat length of n = 13 was determined in one patient. No significant difference was observed in the distribution of genotype and allele between cases and controls.

**Table 3:** The allele distribution of EGFR intron 1 (CA)_n _repeat in ACS patients and controls

	Control (n = 210)		ACS (n = 191)	
		
	n	%^a^	n	%^a^
13	0	0.0	1	0.3
14	2	0.5	3	0.8
15	23	5.5	16	4.2
16	66	15.7	69	18.1
17	28	6.7	24	6.3
18	13	3.1	5	1.3
19	55	13.1	39	10.2
20	180	42.9	172	45.0
21	38	9.0	31	8.1
22	12	2.9	21	5.5
23	3	0.7	1	0.3

^a^Does not add up to 100.0% because of rounding.

To evaluate the effect of the number of (CA)_n _repeats on patients with ACS and controls, we separated the genotypes into two subgroups: both (CA)_n _repeats < 20 and any (CA)_n _repeats ≥ 20. No significant relationship between the number of (CA)_n _repeats of EGFR intron 1 and the risk of ACS was observed (adjusted OR = 0.97, 95% CI: 0.58–1.64, adjusted *P *= 0.911) (Table 2).

### Combined analysis and linkage disequilibrium

When we considered these two polymorphisms together, there was no statistically significant difference between patients with ACS and controls (data not shown).

No statistically significant evidence for linkage disequilibrium between the HER-1 R497K polymorphism and EGFR (CA)_n _repeat polymorphism was found in our study (data not shown).

### Stratified analysis

When the analyses were stratified by hypertension and diabetes mellitus, no significant association was observed between the R497K polymorphism and the risk of ACS (data not shown).

### Multivariable logistic regression analysis

This analysis showed that independent risk factors for ACS were cigarette smoking, total cholesterol, LDL-cholesterol, triglycerides, and R497K (Table 4).

**Table 4:** Results of multivariable logistic regression analysis: final significant variables in equation

Variable	Odds ratio	95% CI	*P*
Smoker	2.63	1.23–5.63	0.013
Total cholesterol	7.29	4.60–11.56	< 0.001
LDL-cholesterol	13.75	6.77–27.93	< 0.001
Triglycerides	6.40	3.46–11.86	< 0.001
R497K	1.72	1.03–2.86	0.039

## Discussion

In this retrospective, hospital-based case-control study, we found that the frequency of *Lys *allele in the ACS group was significantly higher than that in control. Additionally, multivariable logistic regression analysis showed that R497K was an independent risk factor for ACS besides some classical risk factors, such as smoking status, total cholesterol, LDL-cholesterol, and triglycerides. It indicates that *Lys *allele may be a risk factor for ACS in a Chinese population. This finding is in consistent with our hypothesis and suggests that the R497K polymorphism may be used as a genetic susceptibility marker of the ACS.

For the (CA)n repeat in intron 1 of EGFR polymorphism, alleles 20 (42.9%) and 16 (15.7%) were the two most common in a Chinese population in our current study, which was similar to that reported by Liu et al. [34] in a U.S. Asian population (62.9% for allele 20 and 17.4% for allele 16). However, the allelic distribution was reversed in Caucasians and African-Americans, with allele 16 the most common (43.2% and 41.7%, respectively), followed by allele 20 at a much lower frequency (21.0% and 14.3%, respectively). It suggests that the allelic frequencies of the EGFR intron 1 polymorphism might vary among different ethnic groups.

The EGFR, also known as HER-1 or erbB-1, is a member of the human epithelial receptor tyrosine kinase family. The EGFR gene is located on chromosome 7 [15] and contains 26 exons [16]. Exons 1 to 14 code for the extracellular domain. Exon 15 codes for the transmembrane domain, and exons 16 to 26 code for the intracellular domain [35]. Transcription of the EGFR gene is regulated by two enhancer elements. The first is located upstream near the transcription initiation site and the second downstream in intron 1 [36]. The latter contains a characteristic simple sequence repeat (SSR) of CA dinucleotides, the length of which exhibits interethnic differences [34]. In addition, the length of this polymorphism has been observed to correlate inversely with EGFR transcription in both *in vitro *experiments and breast cancer specimens. The longer allele with 21 repeats was shown an 80% reduction of gene expression compared with the shorter allele with 16 repeats [19,20]. In this present study, no significant difference was observed in EGFR intron 1 (CA)_n _repeat polymorphism between cases and controls. When taking HER-1 R497K and EGFR intron 1 (CA)_n _repeat polymorphisms into together, there was no statistically significant difference between patients with ACS and controls as well.

Currently, EGFR is considered as a convergence point in the complex signaling network and regulation of cellular functions, such as cell growth, differentiation, motility, survival and death [37-39]. Thereby, it plays a crucial role in various pathophysiological processes, including atherosclerosis. EGFR has been demonstrated on both intimal smooth muscle cells within human atherosclerotic plaque and cultured rat aortic smooth muscle cells [30-32]. Furthermore, it has previously reported that EGFR is present on rabbit peripheral blood leucocytes, and co-localises with macrophages within lesions of atherosclerosis in the cholesterol-fed rabbit [40], which mediates both chemotactic responses towards monocytes and mitogenic responses towards monocyte-derived macrophages in *vitro*. Transactivation of the EGFR mediates endothelin-1 (a powerful vasoconstrictor, involving in vasospastic diseases such as coronary artery disease) signaling in vascular smooth muscle cells and isolated arteries [41]. This identification heightens the potential importance of EGFR in atherogenesis and other inflammatory disease. A few studies have shown that polymorphism of EGFR was associated with susceptibility of some inflammatory diseases [42,43]. For example, Wang et al. [42] reported that asthmatic patients had a significantly higher incidence of having shorter alleles (≤ 16 CA repeats) compared with control subjects (*P *< 0.05). Martin et al. [43] reported that the frequency of the WT (*Arg*) allele at position R497K in the EGFR gene was significantly higher in ulcerative colitis than in controls (*P *= 0.04).

However, no previous study has shown the relationship between HER-1 R497K and EGFR intron 1 (CA)_n _repeat polymorphisms and patients with ACS. To our knowledge, the present pilot study is the first to investigate the association between them. We found that R497K but not (CA)_n _repeat polymorphism might be used as a genetic susceptibility marker of the ACS. The exact mechanism by which the *Lys *allele may be associated with the risk of ACS is unclear. In 1993, a variant EGFR of an arginine to lysine substitution at codon 497 was identified [17]. Wild-type EGFR (*Arg *allele) differs from mutant EGFR (*Lys *allele) with respect to epithelial proliferation following the administration of EGF and TGF-α in rodents [18]. Thus, it is possible that mutations which influence the function or expression of EGFR (such as attenuating its ligand binding as well as subsequent activation of its downstream effectors) might predispose to the development of ACS.

## Conclusion

To conclude, in the present retrospective study, we found that HER-1 R497K polymorphism was significantly associated with the risk of ACS in a Chinese population. These results suggested that genetic polymorphism of EGFR (arginine →Lysine mutation) might be clinically important in the development and progression of ACS. Nevertheless, there are some limitations in our study, and several questions still should be addressed. For instance, the molecular mechanism by which the EGFR gene polymorphisms are associated with ACS is unknown. It should also be emphasized that other polymorphisms in linkage disequilibrium with the R497K and (CA)_n _repeat may also contribute to variability in EGFR expression. In addition, the inadequate study design such as non-random sampling and a limited sample size should also be considered. Therefore, One needs to explore further, whether EGFR polymorphisms are an independent risk factor or an indirect marker of different genetic and environmental factors. Further studies are required to evaluate haplotype, relationship among genotypes, and the molecular mechanisms by which EGFR is involved in susceptibility to ACS in diverse ethnic populations and larger number of patients.

## Methods

### Study population

The case-control population consisted of 401 adult unrelated Chinese who were selected from the same population living in China (Table 1). A total of 191 ACS patients (112 males and 79 females, mean age: 60.6 ± 10.9), including 102 of whom suffering from unstable angina (UA), 49 from non-ST-segment elevation myocardial infarction (NSTEMI) and 40 from ST-segment elevation myocardial infarction (STEMI), were recruited from West China hospital, Sichuan University between July 2005 and January 2007. The diagnosis of ACS was performed according to the definitions proposed by the European Society of Cardiology and the American College of Cardiology [44]. Exclusion criteria were evidence of significant concomitant diseases, in particular, hemodynamic valvular heart disease, known cardiomyopathy and malignant diseases, as well as a febrile condition.

The control group included 210 healthy volunteers (132 males and 78 females, mean age: 59.4 ± 11.2) who visited the general health check-up division at West China Hospital, Sichuan University. Selection criteria for controls were no history of heart disease or systemic disease and found to be normal by physical examination, electrocardiogram, and echocardiogram. There was no significant difference between patients and control subjects in terms of gender and age distribution. Written informed consent was obtained from all the subjects, and the study was performed with the approval of the ethics committee of Chinese Human Genome.

### Plasma lipid determination

The method was described in our previous study [45]. In brief, venous blood sample was drawn after an overnight fast. Serum cholesterol, HDL-cholesterol, LDL-cholesterol and triglycerides were measured by automated enzymatic methods. All blood lipid samples were examined at Olympus-1000 automated analyzer (Japan).

### DNA extraction and EGFR genotyping

Genomic DNA was extracted from peripheral blood with an extraction kit (Bioteke Corporation; Beijing, China) according to the manufacturer's instructions. R497K in exon 13 genotype was determined by using a PCR-RFLP assay and DNA sequencing analysis. The PCR primers were 5'-TGCTGTGACCCACTCTGTCT-3' (forward) and 5'-CCAGAAGGTTGCACTTGTCC-3' (reverse). The PCR reactions were performed in a total volume of 25 μl containing 100 ng genomic DNA, 20 pM of each primer, 0.2 mM dNTPs, 20 mM Tris-HCl (pH 8.8), 10 mM KCl, 10 Mm (NH4)_2_SO_4_, 2 mM MgSO_4_, 0.1% Triton X-100, and 1 unit of Taq polymerase (New England BioLabs). The PCR cycle conditions consisted of an initial denaturation step at 94°C for 5 min followed by 34 cycles of 30 s at 94°C; 45 s at 60°C and 45 s at 72°C; and a final elongation at 72°C for 10 min. After PCR amplification, the PCR products were digested at 37°C overnight with the restriction enzyme *Mva *I (Fermentas). The digested PCR products, including a single fragment of 155 bp (uncuttable by restriction enzyme) and two fragments of 88 bp and 67 bp (cuttable), were resolved on 2% agarose gel and 8% acrylamide gel respectively, and stained with ethidium bromide for visualization under UV light.

The (CA)_n _repeat polymorphism in intron 1 of the EGFR gene was genotyped by direct-sequencing. The primers were 5'-TTCTCCTCAAAACCCGGAGAC-3' (forward) and 5'-GTCACGAAGCCAGACTCGCT-3' (reverse). PCR conditions were as follows: initial denaturation at 94°C for 5 min; 30 cycles of denaturation at 94°C for 30 s, annealing at 60°C for 30 s, extension at 72°C for 30 s, and final extension of 72°C for 10 min. After PCR amplification, the exact number of the (CA)_n _repeat was confirmed by direct sequencing.

### Statistical analysis

In this exploratory, retrospective study, genotype, allele frequencies and stratified analysis of EGFR were compared between ACS cases and controls using the χ^2 ^test and Fisher's exact test when appropriate. The association between R497K polymorphism and risk of ACS was estimated by computing the ORs and their 95% CIs using logistic regression analyses for crude ORs, adjusted ORs and adjusted *P *value when adjusted for sex and age. Demographic and clinical data between groups were compared by χ^2 ^test or Student's t-test. Hardy-Weinberg equilibrium was tested for with a goodness of fit χ^2^-test with one degree of freedom to compare the observed genotype frequencies among the subjects with the expected genotype frequencies. Multivariable logistic regression analysis was performed on the effect of the EGFR polymorphism and other risk factors for ACS. Statistical significance was assumed at the *P *< 0.05 level. The SPSS statistical software package version 11.5 was used for all of the statistical analysis.

## Authors' contributions

L-BG, BZ and LR conceived of the study, participated in its design, carried out most of the experiments and drafted the manuscript. LZ and Y-SW participated in design of study and helped to draft the manuscript. Y-YW performed DNA extraction. W-BL and M-LL participated in genotyping. X-MP and Y-CC participated in plasma lipid determination. All authors have read and approved the final manuscript.

## Pre-publication history

The pre-publication history for this paper can be accessed here:


